# Incidence, Trend, and Mortality of Human Exposure to Rabies in Yemen, 2011-2017: Observational Study

**DOI:** 10.2196/27623

**Published:** 2021-06-22

**Authors:** Rihana Taher Abdulmoghni, Ahmed Hasan Al-Ward, Khaled Abdullah Al-Moayed, Mohammed Abdullah AL-Amad, Yousef S Khader

**Affiliations:** 1 Yemen Field Epidemiology Training Program Ministry of Public Health and Population Sana'a Yemen; 2 National Rabies Control Program Ministry of Public Health and Population Sana'a Yemen; 3 General Directorate of Disease Control and Surveillance Ministry of Public Health and Population Sana'a Yemen; 4 Department of Community Medicine, Public Health and Family Medicine Faculty of Medicine Jordan University of Science & Technolog Irbid Jordan

**Keywords:** rabies, incidence, trend, mortality

## Abstract

**Background:**

Rabies remains a neglected and poorly controlled disease throughout the developing world, particularly in Africa and Asia, where most human rabies deaths occur.

**Objective:**

This study aimed to describe the epidemiology of rabies exposures, its trend, and its geographical distribution in Yemen.

**Methods:**

Cumulative data from a rabies surveillance system for the period 2011-2017 were obtained from the National Rabies Control Program as paper-based annual reports. Data included the number of persons bitten by a suspected rabid animal, their gender and age, and the result of the animal’s laboratory test. Human cases were defined as those exposed to rabies virus bitten by a suspected rabid animal, exposed to a confirmed rabid animal and then received postexposure prophylaxis (PEP), and deaths occurred after exposure to a confirmed rabid animal after having rabies symptoms during 2011-2017.

**Results:**

From 2011 to 2017, a total of 76,049 persons were bitten by a suspected rabid animal. Of these, 21,927 (28.83%) were exposed to positively confirmed rabid animals and then received PEP, and 295 (0.38%) rabies-related deaths occurred. Of all cases with rabies exposure, 50,882 (66.91%) were males. The most affected age group by animal bites (31,816/76,041, 41.84%), positive exposure (8945/21,927, 40.79%), and rabies deaths (143/295, 48.47%) was 5-14 years. Rabies vaccines and immunoglobulins quantities were least available in 2016 and 2017. The annual incidence rate of exposure to animal bites and rabies exposure was 50 and 14 per 100,000, respectively. The annual mortality rate was 2 per 1,000,000. The highest incidence rate of animal bites was in Dhamar (112 per 100,000) and Ibb (94 per 100,000), whereas the highest incidence of exposed cases was in Amanat Al Asimah (40 per 100,000) and Ibb (37 per 100,000). Mortality rate was the highest in Amanat Al Asimah (6 deaths per 1,000,000) followed by Ibb and Dhamar (4 deaths per 1,000,000 in both).

**Conclusions:**

Rabies remains a worrying health problem in Yemen with higher percentage reported among children and males. Targeting school-age populations by education, communication, and information campaigns about preventive measures is strongly recommended. An electronic system should be introduced to improve reporting. It is important to have a sufficient supply of vaccines and immunoglobulins in control units, especially in the at-risk or impacted governorates. Future studies are suggested to determine incidences and risk factors of disease progression.

## Introduction

Rabies is a zoonotic progressive neurological infection in humans and other mammals caused by the rabies virus, which belongs to the genus *Lyssavirus* (family: Rhabdoviridae) [1]. Over 99% of rabies cases are caused by bites of rabid dogs [1,2]. Rabies is a fatal disease with 2 clinical manifestations: furious (classical or encephalitic) and paralytic. Furious rabies accounts for nearly 80% of cases [3]. Initial symptoms are similar to those of many other illnesses including fever, headache, and general weakness or discomfort. As the disease progresses, more specific symptoms appear and may include insomnia, anxiety, confusion, slight or partial paralysis, excitation, hallucinations, agitation, hypersalivation, difficulty swallowing, and hydrophobia. There is no specific treatment for rabies, and as a result, death usually occurs within days of the onset of these symptoms. However, safe and effective animal and human vaccines are widely available for the prevention and control of rabies [3,4].

Rabies is classified as a poverty-related disease that affects disadvantaged populations with the vast majority of cases being reported in children under the age of 15 years [3]. Rabies remains a neglected disease and is poorly controlled throughout the developing world, particularly in Africa and Asia, where most rabies-related human deaths occur [5]. The disease is reported in 150 countries and territories and causes approximately 60,000 fatalities annually [2]. In Southern and Eastern Mediterranean countries, the burden due to dog-mediated rabies was estimated to be 1875 human deaths and 14,310 disability-adjusted life years per year in Central Asia and 229 human deaths and 1875 disability-adjusted life years per year in the Middle East [3]. However, estimates of burden have always been uncertain due to the lack of reliable data in many countries [2].

In Yemen, canines are the main reservoirs of this disease [4]. The incidence of rabies in Yemen is estimated to be 23 human cases per 1,000,000 population [6]. However, data are usually underestimated due to inadequate diagnosis and underreporting of human rabies in many areas of the country; in particular, laboratory test is only available for a suspected rabid animal, a direct fluorescent antibody test for observing rabies virus proteins in animal tissues, which is performed only in the Central Veterinary Laboratory (CVL). According to the Ministry of Public Health and Population and National Rabies Control Program (NRCP), approximately 50 deaths occur annually, and the number of dog bites was estimated to be 10,017 in 2017 [7]. Rabies postexposure prophylaxis (PEP) is available for humans only, and according to the CVL and NRCP, no vaccines are available for domestic animals [8].

The Rabies Surveillance System was set up in Yemen in the 1980s as a Rabies Control Unit in Sana’a city, and then in Ta’izz and Al Hudaydah. In 1990, the NRCP was established by the Ministry of Public Health and Population under the administration of the primary health care sector. The program now has 26 Rabies Control Units operating in 15 governorates out of the total 22 governorates in Yemen.

The reporting, however, remains a paper-based system. This study aimed to describe the epidemiology of human rabies exposure, its trend, and its geographical distribution in Yemen.

## Methods

Cumulative data as paper-based annual reports from the Rabies Surveillance System for the period from 2011 to 2017 were obtained from the NRCP. Data included the number of persons bitten by a suspected rabid animal, their gender and age, and the result of the animal’s laboratory test in the CVL. Quantities of vaccines and immunoglobulins for each control unit were also included. Human cases were defined as those exposed to rabies virus if bitten by a suspected rabid animal, exposed to a confirmed rabid animal and then received PEP, and deaths occurred after exposure to a confirmed rabid animal and having rabies symptoms during 2011-2017 [3]. Data were entered, cleaned, and analyzed using MS Excel and Epi Info 7.2. Percentages and rates were calculated. Total populations were obtained from the Yemen Central Statistical Organization and used to calculate the incidence rate/100,000 population.

## Results

During the period 2011-2017, a total of 76,049 persons were bitten by a suspected rabid animal. Of those, 21,927 (28.83%) were exposed to positively confirmed rabid animals and then had PEP while 295 (0.38%) rabies deaths occurred. The annual average number of animal bites, exposed cases, and deaths was 10,846, 3132, and 42, respectively.

**Figure 1:**
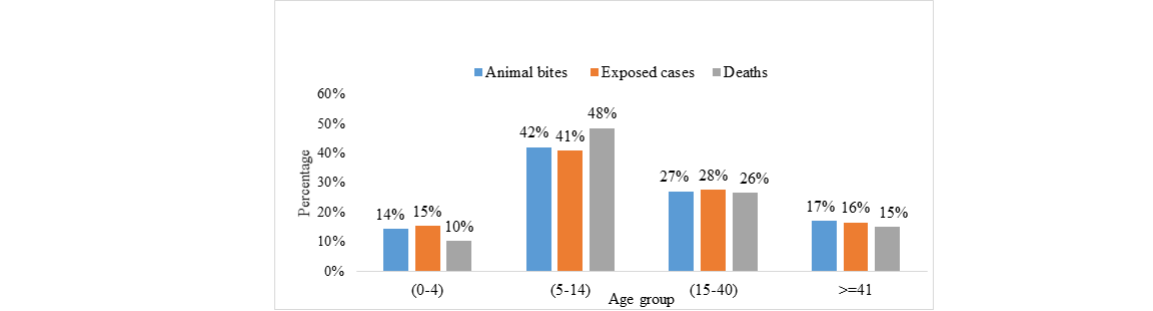
Distribution of animal bites cases, exposed cases, and rabies deaths by age groups in Yemen between 2011 and 2017.

Of all cases with rabies exposure, 50,882/76,049 (66.91%) were males; in particular, children between ages 5 and 14 years were more impacted than other age groups: 31,816/76,041 (41.84%) had animal bites, 8945/21,927 (40.79%) had positive exposure, and 143/295 (48.47%) died (Figure 1).

Available quantities of rabies vaccine and immunoglobulins varied by years; they were the least in 2016 and 2017, with 0 immunoglobulins vials available in 2017, whereas they were the highest in 2013 (data not shown).

The annual incidence rate of exposure to animal bites and rabies exposure was 50 per 100,000 population and 14 per 100,000 population, respectively, and the annual mortality rate was 2 per 1,000,000, population.

**Figure 2:**
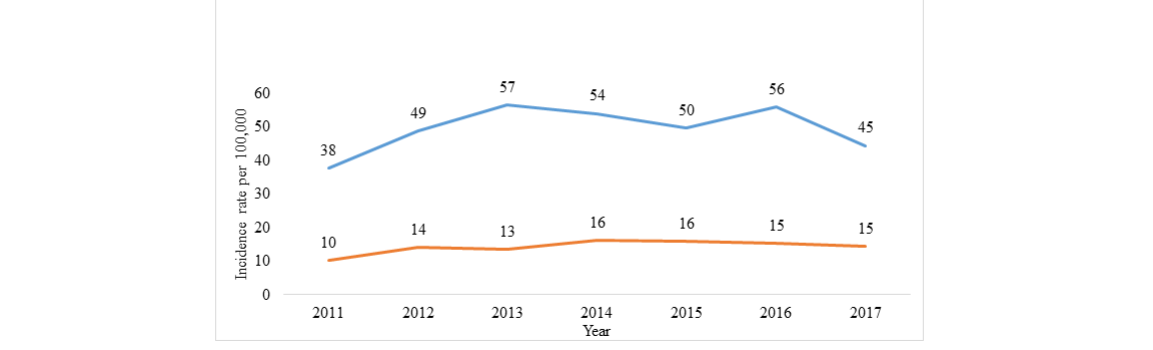
Incidence rate of animal bites and exposed cases in Yemen from 2011 to 2017. Blue line: animal bites/100,000; orange line: exposed cases/100,000.

The incidence rate of animal bites was highest in 2013 and 2016. The incidence rate of rabies exposure increased from 10 per 100,000 in 2011 to 16 per 100,000 (highest rate) in both 2014 and 2015, but then decreased to 15 per 100,000 in both 2016 and 2017 (Figure 2).

The highest number of deaths was reported in 2012 and 2017 (49 deaths in the 2 years). The highest mortality rate was reported in 2012, 2013, and 2017 (Table 1).

**Figure 3:**
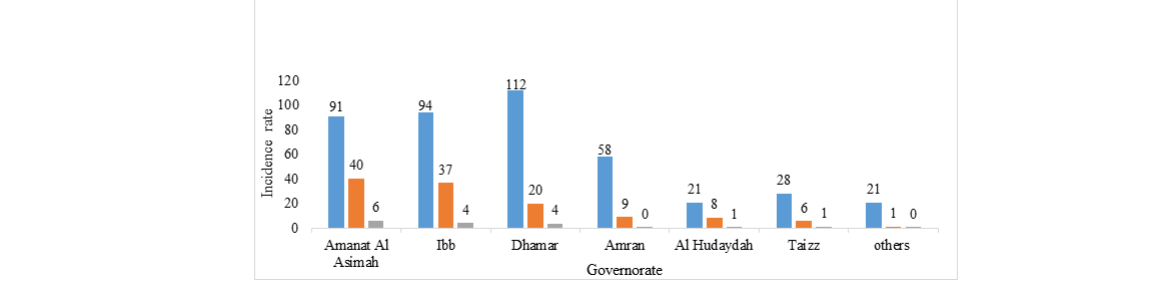
Incidence rate of animal bites, rabies exposed cases, and mortality rate by governorates in Yemen from 2011 to 2017. Blue bars: animal bites/100,000; orange bars: exposed cases/100,000; gray bars: deaths/100,000.

The highest incidence rate of animal bites was in Dhamar and Ibb with 112 and 94 per 100,000 population, respectively, and the highest incidence rate of rabies exposure was in Amanat Al Asimah and Ibb, with 40 and 37 per 100,000 population, respectively. Amanat Al Asimah had the highest mortality rate with 6 deaths per 1,000,000, followed by Ibb and Dhamar with 4 deaths per 1,000,000 population (Figure 3).

**Table 1:** Mortality rates from rabies in Yemen from 2011 to 2017.

Year	Population	Number of deaths	Mortality rate/1,000,000
2011	19,967,804	38	1.9
2012	20,610,108	49	2.4
2013	21,275,006	44	2.1
2014	21,963,378	43	2.0
2015	22,676,140	40	1.8
2016	23,414,249	32	1.4
2017	22,853,036	49	2.1

## Discussion

### Principal Findings

Rabies remains a neglected disease in Yemen. The present study highlights this health problem and provides information about the status of rabies exposure in Yemen.

Our results revealed that during the 7-year period (ie, from 2011 to 2017), the annual incidence rate of exposure to animal bites was 50 per 100,000, which was almost equal to the incidence rate in Oman (46.5 per 100,000 population) [9]. However, the incidence rate in our study was much lower than that in India (1700 per 100,000 population) [10], Iran (13.2 per 1000 population) [11], and Ghana (172 per 100,000 population) [12]. Unfortunately, this result may not reflect the actual status of exposure to animal bites in Yemen due to the underreporting of cases, which is attributed to low community awareness about the disease that in turn leads to very limited number of cases that actually receive proper medical care upon exposure.

The annual incidence rate of human rabies exposure was 14 per 100,000, which was lower than that reported in India, Ethiopia, and Vietnam [13-15], but higher than that in Thailand [16]. This difference is mainly due to variation in study methods employed, or it may well truly reflect the actual situation in these countries. By contrast, the low incidence of rabies exposure in Yemen compared with other counties is likely due to the underreporting of cases.

This study showed that the incidence rate remained unstable from 2011 to 2013, and then it increased from 2014 to 2016, which could be explained by the impact of political conflicts and the war in Yemen during these years. Because the war also affected both nonhealth and agricultural sectors, various measures of the municipality to control the population of street dogs and domestic animals (eg, sterilization) in rural and urban areas collapsed.

The highest number of deaths was in 2012 and 2017 (49 in both). This might be due to the improvement in the reporting system (2012) or to the scarcity of vaccines and lack of immunoglobulins due to siege and war (2017).

Globally, men are at higher risk than women, accounting for 50,882/76,049 (66.91%) of cases. This result was consistent with the findings of a previous study in Yemen [4]. This finding is also in agreement with those of previous studies in Oman, Iran, Ethiopia, India, Bhutan, and Nigeria [9,13,17-20]. The higher rate among men might be due to their increased outdoor activities in comparison to women.

According to the WHO, 40% of individuals impacted by rabies are children aged 4-15 years [21]. In our study, 40.79% (8945/21,927) of exposed individuals fall in this age group. A similar finding was reported in other developing countries such as Iraq and Tanzania [5,22]. The higher rate of being bitten in this age group is likely because children in this age group are more likely to play with, annoy, or approach the biting animals.

Our findings indicate the higher incidence of rabies in the main cities of Yemen, such as Amanat Al Asimah and Ibb. This result may due to improper recording of impacted cases from other governorates with poor health services, as suspected patients tend to visit referral hospitals in nearby governorates with better facilities. In particular, Dhamar recorded a high incidence, which may due to the poor and rural nature of the land.

### Conclusion

Rabies remains a worrying health problem in Yemen, with a higher percentage of cases reported among children and males. The annual incidence of animal bites and rabies exposure was 50 and 14 per 100,000 population, respectively, and the annual mortality rate was 2 per 1000,000 population. Education, communication, and information campaigns about preventive measures by targeting school-age populations are strongly recommended. An electronic system should be introduced to improve reporting. It is important to have a sufficient supply of vaccines and immunoglobulins in control units, especially in the at-risk or impacted governorates. Moreover, vaccinating canines to avoid animal-to-human transmission are necessary. Finally, future studies are suggested to determine incidences and risk factors of disease progression.

## Abbreviations

CVL: Central Veterinary Laboratory
NRCP: National Rabies Control Program
PEP: postexposure prophylaxis
